# Obliteration of the Cava Inferior

**Published:** 1830-10-01

**Authors:** 


### XLVII.

### HOPITAL DE LA PITIE.

### Obliteration of the Cava Inferior.

The proofs of the disease in the following case are not yet verified by dissection; but the case itself is scarcely less interesting on that account.

*Case*. Lefevre, a porter, aged 40 years, of rather weakly constitution, entered the hospital on the 8th of May, 1830, having been ill fifteen days. Eight months previously, he had been in La

CHARITÉ for fracture of the *right* fibula, during a period of five months, and experienced no constitutional indisposition while there. He was never afterwards able to walk without crutches. For the four months after leaving the hospital, he experienced often a diminution of appetite, chills, &c. and one month before coming to LA PITIÉ, he fell down a staircase, and struck the right side of his chest against the wall, which was followed by some pain in that part, and slight cough. Two weeks before he came under the observation of M. Louis, the patient felt pain in the lower and inner side of the right thigh, which gradually extended to the groin, with swelling of the whole limb. There was nothing of the kind in the other extremity till a week afterwards, when it also swelled.

*9th May.* Both lower extremities are œdematous, but the *left* much more so than the *right*. There was neither redness nor hardness, nor pain along the internal surface of the limbs—immobility, without paralysis, existed in the *left* thigh for two days past. In the groin of that side there were two red bands—and the veins of both hypogastric regions were very much developed those of the thighs not at all apparent. There was no perceptible tumour in the abdomen; but pressure on the lower part could not be borne. There was no fever, though the pulse was 100 in the minute. There was a little embarrassment in the breathing; but the respiration was good, as was the sound on percussion throughout the chest. Venesectio ad ℥xij.—diluents. There was a slightly inflammatory crust on the blood. *10th May.* He was considerably relieved by the bleeding—the pulse fell to 95—there was less malaise, but a greater elevation of temperature. Some tincture of digitalis and nitre with the ptisans. In the evening a rigor, followed by heat and perspiration. *11th May.* Heat natural—pulse 88—left thigh softer—red bands still continue in the groin, but less marked—the abdominal veins more salient. Venesection to ten ounces. The blood was not inflamed. There was another rigor, with fever and perspiration this day. 12th. The epigastric veins still more enlarged than natural, and the blood evidently circulated in a retrograde direction—that is, from the trunks towards the branches, as ascertained by pressure. There was no particular alteration till the *16th May*, except the disappearance of the febrile paroxysms above-mentioned, and the complaint of a pain in the left hypochondrium, and in the lower part of the chest of the same side. The patient coughed and expectorated a little; and on examination, there was no sound in the lower third of the left side of the thorax. From this time till he quitted the hospital, on the 7th of August—that is during three months, the following phenomena were observed. The œdema of the lower extremities progressively diminished, and, by the 6th of June, was scarcely perceptible in the right member, but more marked in the *left*. By the middle of July, there was no swelling in either limb. The state of the veins changed with that of the swelling. On the 26th of May, the right epigastric vein was much more developed than the left—by the 6th of June, both veins were considerably diminished. By the 14th, the right was nearly effaced—on the 15th, pressure made on the track of neither vessel caused turgescence. When he left the hospital, no trace of the epigastric veins on either side was visible. Leeches had been applied to the left side of the chest twice, soon after which the cough diminished, but the sound was not clear till the 6th June. On this day, the patient complained of pain in the *right* side of the chest, with an augmentation of the cough, but without any loss of sound in the corresponding part. This pain ceased on the 19th. There was but little thirst all this time, and the appetite never entirely failed though it was very much impaired. He left the hospital in a very enfeebled condition, not being able to walk even with the aid of crutches.

M. Louis thinks that the phenomena above detailed—and especially the retrograde circulation in the enlarged epigastric veins, prove incontestibly that an obstruction had formed in the inferior cava. The ultimate diminution of these vessels, he thinks, is attributable either to removal of the obstacle in the cava, or to a more deep-seated collateral circulation having been established. We are inclined to agree with the talented physician of LA PITIÉ, and, as the poor man seems to be in no very promising condition of health, it is not improbable that the issue of the case, and the diagnosis of the disease, will be put to the test of *post-mortem* examination.—HEBDOMADAIRE.

---

